# Supporting the Advancement of a National Agenda for Pediatric Healthcare Reform: A multi-year Evaluation of a Leadership Education in Neurodevelopmental and Related Disabilities Program

**DOI:** 10.1007/s10995-025-04040-7

**Published:** 2025-01-23

**Authors:** Caitlin Koob, Sarah F. Griffin, Mackenzie Stuenkel, Kathleen B. Cartmell, Kerry Sease

**Affiliations:** 1 Department of Public Health Sciences, Clemson University, 501 Edwards Hall, Clemson, SC 29634 USA; 2 Prisma Health Children’s Hospital-Upstate, Greenville, SC USA; 3 Vanderbilt University Medical Center, Nashville, TN USA; 4 Institute for Advancement of Community Health, Furman University, Greenville, SC USA

**Keywords:** Interdisciplinary Training, Leadership Education, Children and Youth with Special Healthcare Needs, Health care Provider Education, Pediatric Healthcare

## Abstract

**Objectives:**

To evaluate the implementation and sustainability of the effect of a 1-year Leadership in Education for Neurodevelopmental and related Disabilities (LEND) program in a southeastern state, and to examine its impact on advancing the Maternal Child Health Bureau’s (MCHB) *Blueprint for Change*—a national agenda for pediatric healthcare reform.

**Methods:**

This study applies the Exploration, Preparation, Implementation, and Sustainment (EPIS) framework to rigorously evaluate LEND implementation and impact between 2018 and 2022. In-depth interviews (*N* = 24) were conducted among long-term (1-year) LEND trainees, via Zoom, in a southeastern state. A hybrid approach of deductive and inductive thematic analysis was conducted to identify emergent patterns and themes from trainees’ experiences, related to the EPIS constructs and national priorities.

**Results:**

*Exploration and Preparation.* Trainees identified insights from multidisciplinary discussions and family panels as key facilitators to their development. *Implementation.* Trainees reported growth in confidence and communication and improving their service delivery, including implementation of a collaborative approach to patient care, family-centered care, and occasionally facilitating their obtainment of leadership positions. Trainees also reported systemic barriers to implementation, including time and financial constraints. *Sustainability.* Trainees identify their experienced shift in mindset and statewide connections as drivers for sustained change, with suggestions for follow-up events and networking opportunities to enhance the effect of LEND training.

**Conclusions for Practice:**

These results may inform LEND objectives to enhance the statewide network and to advance a national framework for prioritizing family well-being and quality of life and access to services.

**Supplementary Information:**

The online version contains supplementary material available at 10.1007/s10995-025-04040-7.

## Significance

Leadership in Education for Neurodevelopmental and Related Disabilities (LEND) is a graduate-level, interdisciplinary training program, with 60 sites funded by the Maternal Child Health Bureau nationwide. LEND is well-positioned to advance the *Blueprint for Change*, a national agenda for pediatric healthcare reform among children with special healthcare needs. This study’s LEND program has not been rigorously evaluated since its inception in 2011. This study seeks to evaluate the perceived sustainability of the effect of LEND training and examine its congruence with the priority areas of the *Blueprint for Change*, within the Exploration, Preparation, Implementation, and Sustainment framework.

**Supplementary Information:**

The online version contains supplementary material available at 10.1007/s10995-025-04040-7.

## Introduction

Children and youth with special health care needs (CYSHCN) comprise approximately 20% of the pediatric population in the United States and account for approximately 50% of pediatric healthcare expenditures (*Children with Special Health Care Needs: NSCH Data Brief*,* July 2020*, n.d.; Coller et al., 2020; Kuo et al., 2022; Warren et al., 2022). While CYSHCN disproportionally rely on the healthcare system, approximately 85% of CYSHCN nationwide do *not* receive services in a well-functioning healthcare system, experiencing persistent unmet health needs and increased family burden (Caicedo, 2014; *Children with Special Health Care Needs: NSCH Data Brief*,* July 2020*, n.d.; Coller et al., 2020; Hoover et al., 2022; Pilapil et al., 2017; Van Cleave et al., 2022).

Critical services gaps have led the Maternal Child Health Bureau (MCHB) to support CYSHCN and their families’ involvement in research and clinical partnerships (Warren et al., 2022). Cross-sector collaborations between CYSHCN, their families, providers, and advocates are crucial to improving population health among CYSHCN (Franz et al., 2021; Hoover et al., 2022). MCHB leverages the American Academy of Pediatrics’ *Blueprint for Change* as a new lens of healthcare for CYSHCN to recognize professional-family partnerships as fundamental to large-scale change, advocating for training curricula that involve patient and family partners (Brown et al., 2022; Coleman et al., 2022; McLellan et al., 2022).

The Leadership Education in Neurodevelopmental and Related Disabilities (LEND) program is an interdisciplinary training program, funded by MCHB, to improve the health of CYSHCN and their families through a statewide network of healthcare providers, legal professionals, educators, individuals with disabilities, and their families (Sharma et al., 2014). LEND is implemented at the graduate-level to address education-related gaps among healthcare providers who work with CYSHCN, with 60 training sites nationwide (Bishop et al., 2022, 2023; *Leadership Education in Neurodevelopmental and Related Disabilities (LEND) Fact Sheet*, n.d.; McLellan et al., 2022; Rosenberg et al., 2015).

The purpose of this study is to explore the relationship between LEND program implementation and sustainability over time on practice- and patient-level outcomes. State-level evaluations of unmet needs among CYSHCN and their families may provide regional context and improve family-centered services in achieving MCHB objectives (Camelo Castillo et al., 2022). There are several LEND training tracks, with long-term trainees participating in a 1-year, > 300-hour curriculum. However, this study’s LEND program has not been rigorously evaluated since its inception in 2011, with gaps in implementation and sustainability of educational outcomes on interdisciplinary providers (Bishop et al., 2022, 2023; Edwards et al., 2014; *Leadership Education in Neurodevelopmental and Related Disabilities (LEND) Fact Sheet*, n.d.). Therefore, with a southeastern state’s LEND program serving as a case study to improve the rigor of evaluating LEND programs nationwide, this study aims to (1) compare perceived sustainability of effect of LEND training, as measured by comparisons in CYSHCN outcomes and their site delivery, among long-term trainees, and (2) identify factors that may affect the implementation of LEND training, as aligned with national priorities among CYSHCN (Brown et al., 2022).

## Methods

### Theoretical Constructs

The life course perspective theorizes the sequence of age-sensitive events that a child experiences influence their longitudinal health outcomes, including relational and physical environments (Bengtson & Allen, 1993; Edwards et al., 2014). With guidance from MCHB, the LEND curriculum follows the life course perspective, highlighting the potential for providers to implement evidence-based strategies into their service delivery and enhance their patients’ long-term health outcomes because of their training. Likewise, this study adopts the life course perspective (Edwards et al., 2014).

### Evaluation Framework

This study applies the Exploration, Preparation, Implementation, Sustainment (EPIS) framework to rigorously evaluate this LEND program. The EPIS framework is a dynamic, cyclical evaluation framework that contains well-defined phases of (1) Exploration, (2) Preparation, (3) Implementation, and (4) Sustainability and examines the implementation of evidence-based practices across a variety of settings, including community and allied health sectors (Moullin et al., 2019). In this study, EPIS guided conceptualization, interview questions, and a preliminary codebook for thematic analysis to understand how LEND concepts are applied into practice among those who have completed long-term training and to understand barriers and facilitators to sustainable changes in service delivery.

### Operationalization of the Blueprint for Change

This study focuses on Critical Areas 2 and 3 of the *Blueprint for Change—* “family and child well-being and quality of life” and “access to services” (McLellan et al., 2022). Priority areas guided interview question development and were coded to drive interpretation of LEND’s role in advancing national agendas among interdisciplinary healthcare providers (Appendices A and B). These priorities align with the LEND mission statement and are well-supported by LEND training curriculum (Bishop et al., 2023; Edwards et al., 2014; *Leadership Education in Neurodevelopmental and Related Disabilities (LEND) Fact Sheet*, n.d.; McLellan et al., 2022).

### Study Design

This study utilizes a qualitative retrospective, longitudinal design to engage LEND long-term trainees from the past five cohorts.

### Sample Population

Of statewide applicants, LEND invites two healthcare providers per discipline to participate as long-term trainees annually. Disciplines include developmental-behavioral pediatricians, occupational and physical therapists, speech language pathologists, audiologists, mental health counselors, social workers, and genetic counselors. Long-term trainees complete a 1-year, > 300-hour training track. LEND Leadership hold senior-level faculty, advocacy, and clinical positions statewide and select a subset of qualified applicants to participate as long-term trainees.

For this retrospective study, eligible participants (1) currently practice within their discipline and (2) successfully completed this state’s LEND program as a long-term trainee from 2018 to 2022. LEND Leadership facilitated recruitment efforts by providing eligible graduates’ email addresses. In total, 79 participants were contacted, with a 31% response rate. A systematic, convenience sampling method was used to recruit from each training year and discipline for a representative sample (Table 1).

**Table 1:** Study participants by training year

LEND Training Year	Number of participants	Discipline(s) included (Number of participants per discipline)
FY18	5	Psychology* (2), Nurse practitioner (1), Speech therapy (1), Physical therapy (1)
FY19	5	Physician (1), Psychology* (2), Physical therapy (1), Occupational therapy (1)
FY20	4	Nursing (1), Psychometrist (1), Occupational therapy (1), Support coordinator for advocacy organization (1)
FY21	5	Social work (1), Physician (1), Speech therapy (1), Physical therapy (1), Psychology* (1)
FY22	5	Nursing (1), Social work (2), Psychology* (1), Occupational therapy (1)

*Note: Those who work in Psychology involved school and healthcare settings

### Data Collection and Analysis

Upon agreeance, participants were scheduled for virtual interviews via Zoom, due to LEND’s statewide reach (Archibald et al., 2019). Interviews (*N* = 24) were conducted from February 23 to July 5, 2023. Participants provided verbal consent prior to the interview, and each interview was recorded and transcribed by an independent transcription service. Interviews lasted between 13 min and 44 min, with an average duration of 23 min. Incentives of $20 gift cards were provided.

Qualitative data analysis was conducted using ATLAS.ti Web (Paulus & Lester, 2016). An initial codebook was developed based on literature review and clinical experience among this research team and was referenced throughout the process to ensure consistency among coders (Appendix B). Transcripts were analyzed by this research team, with four of 15 transcripts double coded and compared by independent coders. Coders identified and discussed discrepancies to reach mutual consensus. Analysis followed a deductive-inductive thematic analysis by adapting the initial codebook based on emergent findings (Bingham, A. J., & Witkowsky, P., 2022). Coders refined the existing codebook to best represent the data and reach final consensus on thematic findings. Illustrative quotes are presented in this analysis (Chun Tie et al., 2019; Vanover, Charles; Mihas, Paul; Saldana, Johnny, n.d.).

Preliminary findings were discussed, addressing potential discrepancies in data interpretation, with LEND Leadership to reduce bias and participate in member checking. Final themes were disseminated among LEND Leadership to inform future objectives and program implementation.

Research ethical issues including informed consent, anonymity, and participant confidentiality were carefully addressed throughout the study process. This study was approved and deemed exempt by the Clemson University Institutional Review Board.

## Results

**Fig. 1:**
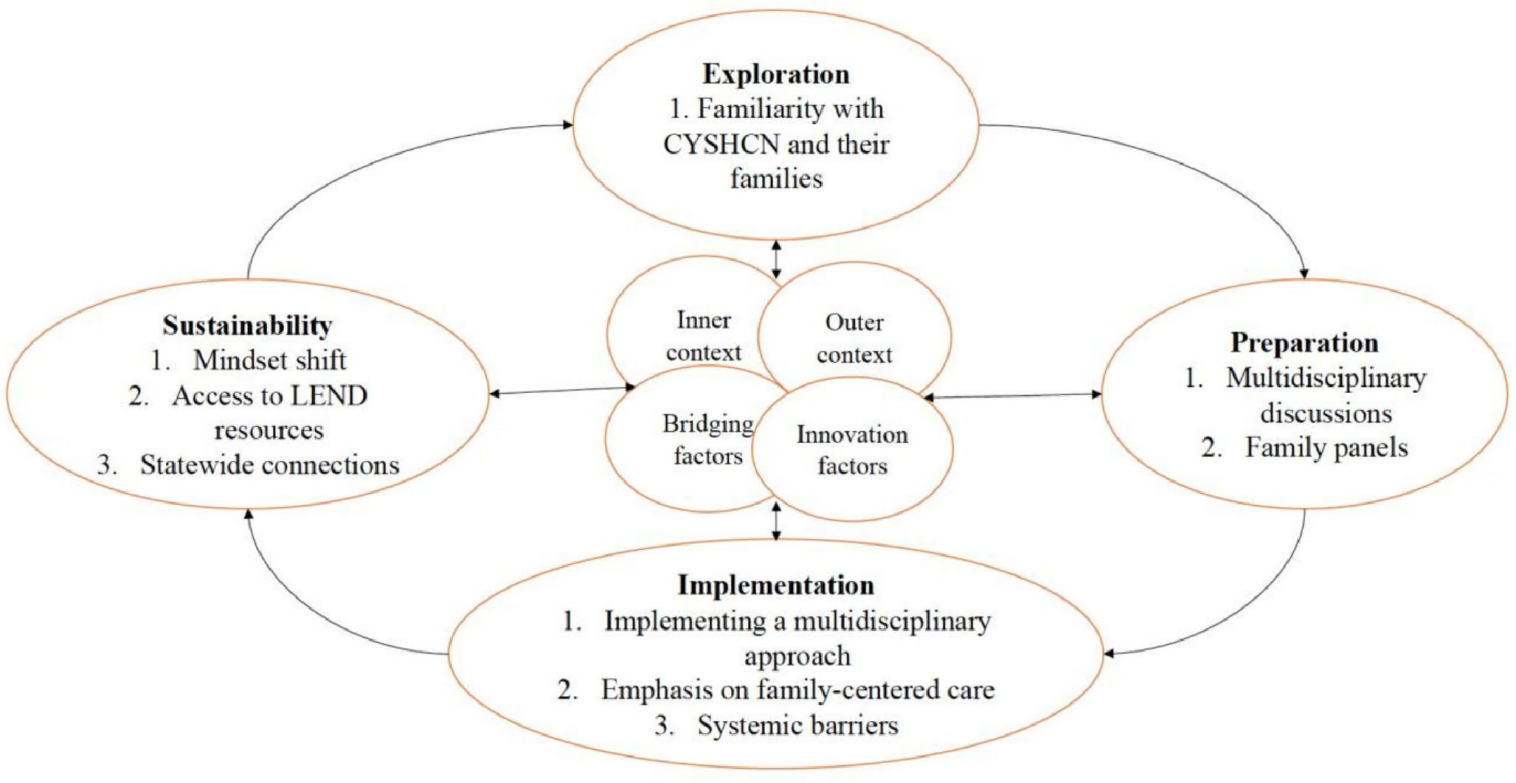
Interconnections between final themes and EPIS constructs

Results were consistent across LEND cohorts, regardless of provider type and/or practice setting. Providers described discipline-specific examples of their application of LEND principles to their service delivery; however, providers’ descriptions of learned concepts were consistent. Table 2 summarizes findings by EPIS construct. Eight themes were constructed and are presented along the EPIS framework (Fig. 1). (Bingham, A. J., & Witkowsky, P., 2022; Moullin et al., 2019).

**Table 2:** Summary of key findings, organized by the Exploration, Preparation, implementation, and Sustainment Framework domains and constructs (Moullin et al., 2019)

EPIS Phases		
**Exploration** In this phase, stakeholders consider the existing needs of the population and work to identify evidence-based practices to address population needs.	**Facilitators**	**Barriers**
	Work with CYSHCN prior to LEND Familiar with colleagues who have participated in LEND Eager to learn and develop leadership skills	
**Preparation** This phase involves planning implementation supports and developing a climate where the proposed evidence-based practice is accepted.	**Facilitators**	**Barriers**
	Learning from didactic modules and patient/family advocates Practice addressing patient needs in multidisciplinary discussions	Intimidated by interdisciplinary collaboration
**Implementation** In this phase, evidence-based practice is initiated in systems and organizations.	**Facilitators**	**Barriers**
	Changes in positionality Improved confidence Improved communication	Systemic barriers, including time and financial constraints
**Sustainment**: Additional contributing factors are ongoing to support continued evidence-based practice delivery, with appropriate adaptations as needed.	**Facilitators**	**Barriers**
	Obtainment of leadership and advocacy positions Statewide networks established among cohorts Continual use of provided resources Mindset shifts among providers	COVID-19 limitations Entirely virtual program delivery Lack of follow-up Participants moved states following LEND
**Contributing factors within additional EPIS constructs**		
	**Facilitators**	**Barriers**
**Leadership** Outer context, inner context	Improved communication Improved confidence Eager to implement evidence-based practices and improve service delivery	Re-establishing statewide connections for participants who moved
**Innovation/Evidence-based practice fit** Outer context, inner context, bridging factors, innovation factors	Support from organizational leadership Family panels Education from self-advocates of individuals with disabilities Evidence-base of a multidisciplinary model among CYSHCN Partnership between community-based organizations, health systems, and academic institutions Experience among LEND leadership	Limited by systemic time and financial constraints
**Service Environment/** **Policies** Outer context	Understanding of family factors and SDOH impact on patient outcomes Understanding of payment policies Collaborative, interdisciplinary environment Support from organization	Family-centered care limited by time constraints Patient access to services limited by insurance policies
**Funding/Contracting** Outer context	Support from MCHB National network of LEND trainees	
**Organizational characteristics &** **Inter-organizational environment and networks** Outer context, inner context, bridging factors	Statewide connections established through LEND Local providers implementation of collaborative care models Community-academic partnerships	Maintaining/re-establishing provider networks
**Patient/client characteristics** Outer context, inner context	Variety of patient needs and symptomology Patient-centered care	
**Patient/client advocacy** Outer context	Improvements in referral practices, resource acquisition Improvements in patient/family satisfaction	Limited by systemic constraints Requires policy-level change

### Exploration

The “Exploration” phase identifies existing needs among CYSHCN and investigates LEND as an evidence-based practice to address the population’s needs (Moullin et al., 2019). Trainees reported *familiarity with CYSHCN and their families* from coursework and clinical experience, prior to LEND (Table 3). Trainees were encouraged to apply to LEND based on their knowledge of existing needs among CYSHCN. Some participants reported LEND exposure from their colleagues, as some organizations’ graduate-level fellowships recommended LEND involvement and affected their decision to pursue long-term training.

**Table 3:** Quotes related to *Exploration* and *Preparation* of LEND training among long-term trainees

Construct	Theme	Illustrative Quote
Exploration	Familiarity with CYSHCN and their families	“[LEND is] a multidisciplinary education program for professionals in a variety of healthcare settings. To learn specifically about neurodevelopmental disabilities, as well as how to interact with multi-disciplinary care in order to improve outcomes for patients with neurodevelopmental disabilities.” - FY18_PT “I encourage individuals that work with families and children who have disabilities to become more familiar. So I say a great way to do this is to get knowledge using the LEND program, if you apply and get accepted, so I do encourage it to a lot of professionals.”– FY22_MH
Preparation	Multidiscip-linary discussions	“There would have been no other part of my training experience I don’t think where I would have interfaced with developmental pediatricians the way I did in LEND… If I’m going to explain autism this way…but maybe a speech language pathologist is going to think about it in a different way…or maybe I’m more behavioral and then they’re going to take it from a different perspective.” - FY18_OP PSYCH “I have been exposed to a lot of multidisciplinary clinics in my training program, like in my graduate program that was an emphasis, but I don’t think I ever actually like got to know personally, as large a group of multidisciplinary providers as I did in LEND. That was huge, just meeting friends and future colleagues, and that definitely helped me apply the multidisciplinary model a lot better.” - FY18_ PSYCH
	Family panels	“We did attend [a] family panel and listened to family perspective of care that they’re receiving. You always want to remember, there’s a family. It’s bigger than just the patient you’re seeing. It’s a whole family and they’re just trying to get the best for their children.” - FY20_NP “…hearing them talk about the day-to-day struggles of just being able to get whether it was equipment or transportation, like getting kids in the car to go or just kind of the emotional toll of things on families, and how that just could impact them getting to therapies or them even under some of the health literacy components too.” - FY19_OT “Sometimes, I can go through the motions too much and just forget about each individual family’s experiences. I look back to the family panels and remember the things that they shared that not necessarily negative, but just their constructive criticism of when they got their diagnosis or when the child received their diagnosis and what that felt like for them.” - FY19_IP PSYCH

I encourage individuals that work with families and children who have disabilities to become more familiar. So I say a great way to do this is to get knowledge using the LEND program.

–FY22_MH

Trainees unanimously reported participating with some baseline knowledge of LEND and identified LEND as a mechanism for implementing evidence-based practice. Trainees explained intrinsic motivation and leadership capacity to advocate for unmet needs of CYSHCN at a patient- and practice-level.

### Preparation

The “Preparation” phase involves planning to implement LEND principles’ into providers’ service delivery, including reflection of past experiences and how they can improve healthcare quality for CYSHCN (Bengtson & Allen, 1993; Moullin et al., 2019). Most trainees cited *multidisciplinary discussions* and *family panels* as facilitators to their development (Table 3). Regarding multidisciplinary discussions, trainees described the value in translating their graduate-level training within a collaborative learning environment, simulating their clinical settings. Many trainees credited the organizational characteristics, including support for interdisciplinary care and continuing education, of their clinical settings when discussing their ability to participate in LEND.

Family panels within LEND curriculum were often cited as the *most* helpful in learning about family perspectives and informed meaningful changes in providers’ service delivery. Trainees considered their service delivery and opportunities for provider-level change after hearing the experiences of parents of CYSHCN and adults with various disabilities who serve as self-advocates.

“Sometimes, I can go through the motions too much…I look back to the family panels and remember the things that they shared that not necessarily negative, but just their constructive criticism of when they got their diagnosis or when the child received their diagnosis and what that felt like for them.” -FY19_IP PSYCH.

While providers discussed a more comprehensive understanding of CYSHCN and their families’ daily lives, some providers considered their mindset adjustments and approaches to family-centered care—translating their new clinical training to service delivery.

### Implementation

The “Implementation” phase occurs when LEND concepts are initiated within the healthcare system, following trainees’ completion of the training (Moullin et al., 2019). Facilitators and challenges to implementation were attributed to (1) *implementing a collaborative approach to patient care*, (2) *emphasis on family-centered care*, including social drivers of health (SDOH), and (3) *systemic barriers* (Table 4).

Trainees explained their improved understanding and ability to implement a multi-disciplinary approach across practice settings. While trainees reported didactic understanding of this team-based model from graduate school, LEND provided an opportunity to practice within a supportive learning environment and foster interdisciplinary relationships.

“In my graduate program [multidisciplinary care] was an emphasis, but I don’t think I ever actually like got to know personally, as large a group of multidisciplinary providers as I did in LEND. That was huge…and that definitely helped me apply the multidisciplinary model a lot better.” -FY18_ PSYCH.

**Fig. 2:**
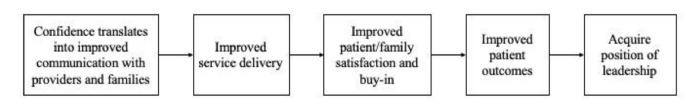
Progression of the effect of LEND training on trainees’ confidence and communication skills and their impact on patients and families’ health well-being. *Note*: Professional state network facilitates connections between disciplines and/or providers across settings

Further, trainees consistently reported *individual growth* through their LEND training. Trainees most frequently reported improved *confidence* and *communication* with interdisciplinary providers, CYSHCN, and their families (Fig. 2). As a result, trainees reportedly experienced improvements in their service delivery and their patient and family satisfaction, permeating into family-level outcomes. Over time, some trainees discussed acquiring leadership positions to instill confidence within their organizations and continue this cycle at an organizational level—translating individual change to the *service environment and broader networks*. As a result, physicians and other interdisciplinary providers described their reliance on other providers as part of a broader care team to support patient care plans and optimal health outcomes, since their LEND training.

As a byproduct of individual-level growth, long-term trainees reported improvements in patient-level advocacy, including resource acquisition, referral appropriateness, and communication on behalf of patients and their families to providers who did not participate in LEND. Some trainees reported acquiring leadership and/or advocacy positions in collaborative settings, establishing themselves as leaders in their practice and local regions. One provider described her role in a national advocacy position for systemic change. However, some trainees identified the vast need for higher-level advocacy as an overwhelming barrier to encouraging large-scale change among providers.

In providers, I think a lot of it is like, oh… that seems like a huge thing…But everybody knows how to do it and then nobody touches it, and it just continues to be the same system.

–FY18_PSYCH.

Still, trainees described the crucial effect of LEND training on their family-centered care practices. Per trainees, *family-centered care includes concepts representative of humility*,* open-mindedness*,* and advocacy*. Trainees reported adapting their service delivery through improved understanding of SDOH and their impact on family functioning and healthcare experiences. These perceptions were reported among trainees in inpatient, outpatient, and school-based settings alike. Some providers described specific changes to their clinical recommendations to better consider CYSHCN and their families’ burden of navigating SDOH and personal constraints to access referred services. In some cases, trainees partnered with families to address previously undetected concerns and tailored their treatment plans accordingly.

“Just me gaining a little bit more humility of that these families and the child’s lives are very complicated, and there’s sometimes going to be competing interests for time, energy, financial capacity. That’s part of my job, is to help the family…not give them recommendations or suggestions that are not feasible or achievable.” -FY18_PSYCH.

Despite strong awareness of SDOH and contextual factors that inhibit patients’ healthcare access, *systemic barriers*—related to financial and time constraints—persist across settings. These barriers prevented trainees from fully integrating concepts learned during LEND into their practices.

“You’re seeing these families get turned away…That’s when it really starts, at least for me, eating you as a therapist. And then you start digging even deeper and realizing that system is so broken. It’s insane.” -FY19_PT.

In these cases, trainees reported being unable to overcome systemic barriers despite best efforts. Still, trainees repeatedly described persistently advocating for patients within patient-provider relationships and clinics within their respective health systems to overcome barriers as able.

**Table 4:** Quotes by subtheme of *implementation* of LEND objectives into providers’ service delivery

Subtheme	Illustrative Quotes
Implementing a collaborative approach to patient care	“It’s definitely opened my perspective to the importance of having a full interdisciplinary team. I think it’s made me realize how many different things each provider, each different role in a child’s care is doing behind the scenes.” - FY22_RN “I’ve improved referrals to other specialties. I think it’s helped me to just be more confident about interdisciplinary care…Even just making those connections [through LEND], I feel more comfortable personally reaching out…I feel like it helped me break down those barriers and not feel like an island where I’m at. Just made me feel like I can reach out to them more without as much intimidation.” - FY21_SLP “I guess it just gave me more of understanding of what other disciplines and what they could provide– like, the kiddos that I was already seeing. Like, if you got any referrals, feeling like you could know where to direct families for different types of care and help.” - FY18_SLP
Individual growth	“I think increased my ability to be able to communicate with doctors. I work at a clinic that has speech, PT and OT, and so I’m constantly collaborating with other specialties. I think I feel more competent because of the LEND program doing that.” - FY19_OT “I collaborate with, other therapists or, to kind of get more information and see what they think and because they know that they think and because they know the child, they’ve seen them a lot more frequently than I did. So, I collaborate a lot more frequently than I did.” - FY21_DBP
Emphasis on family-centered care	“I think it’s helpful to kind of see a lot of the other challenges that the family might be facing… then think about all the other things that the family might have going on that might affect this, and how that’s gonna affect if they’re sick and don’t feel well, they’re gonna miss their therapy appointments, and how detrimental actually can be. And just like the cascade that one will hiccup for maybe a neurotypical child wouldn’t have.” - FY22_RN “I think I’m more open-minded, probably, because of my LEND training, and it’s made probably me more sympathetic and empathetic to what families go to on a day to day basis when they have a child that is neurodevelopmentally different.” - FY22_SCHOOL “Just me gaining a little bit more humility of that these families and the child’s lives are very complicated, and there’s sometimes going to be competing interests for time, energy, financial capacity. That’s part of my job, is to help the family think about that and not overwhelm them or not give them recommendations or suggestions that are not feasible or achievable because then that’s just gonna create a gap where they’re gonna feel like they’re somehow failing something which is not on them.” - FY18_PSYCH “I do have families of color, or a black parent talk to me about how their autistic child might grow up with again, additional barriers. I’ve connected them with different programs on policing…that was a specific request from a parent…how to help prepare a black autistic child for if they ever encounter a police officer.” - FY19_IP PSYCH “It makes people like you, and it increases buy in to therapy if you can explain it in a way that’s relevant to them, they’re much more likely to bring a kid back, they’re much more likely to do what you tell them to. They’re much more likely to talk about you in a positive manner to other people, which helps their child get better quality of care and helps get better quality of care to all the other children in the community.” - FY18_PT “I think I’m able to explain the why a lot more… I think something that LEND gave me that I didn’t have before was just really, it’s like I knew it, but I wasn’t really practicing it of like our parents are all on different levels. Their accessibility levels. You have to change how you give information to them so they can receive it.” - FY21_SLP “That’s just going to create a gap where they’re going to feel like they’re somehow failing something which is not on them…it’s given me a better understanding and respect for the broader systemic things that are impacting individuals’ experiences, whether it be those pieces about equity or racism, but also just healthcare systems…and all the other things that impact someone’s access.” - FY18_OP PSYCH
Systemic barriers	“You’re seeing these families get turned away, or you’re requesting equipment and equipment getting denied, and you’re like, why is this necessary piece of equipment getting denied for my child? That’s when it really starts, at least for me, eating you as a therapist. And then you start digging even deeper and realizing that system is so broken. It’s insane.” - FY19_PT “I’m sure there are so many things that could be done to prevent children from falling through the cracks…I wish there was some way for [referrals] to come back to me, it’d be like, well, we’ve reached out x, y, and z number of times, and the family has declined services. Then I could at least reach out to the family and be like, hey, why are you not making an appointment for this speech evaluation.” - FY18_NP “It’s just not sustainable in healthcare system so there’s a lot of limitations in that way. I guess to your question of how easy or how hard, I still think I’m probably figuring out how to do that best within all the limitations around.” - FY18_PhD “In providers, I think a lot of it is like, oh, like I don’t even know how to, that seems like a huge thing that I just don’t want to touch. But everybody knows how to do it and then nobody touches it, and it just continues to be the same system.”**-** FY18_PSYCH
Time constraints	“I feel like it’s not specific to my sites, but just waitlists are so out of control.” - FY22_OT “We have 15-minute time slots. Then you have four patients in an hour, but your 8:20 patient shows up at 8:35 so that your whole morning is off…I wish so badly I could really go through things so much more thoroughly than I in all actuality I’m ever able to…We’re getting 10 to 15-minute windows of patients, so really trying to prioritize like what do I talk about.” - FY18_NP
Financial constraints	“It’s hard to serve the population that I want to serve the most, because they are the population on Medicaid. It’s not sustainable to only make $30 of reimbursement for $150 eval. All those kids deserve health care…I can never own a business because I’d run it straight into the ground… That’s the biggest one and the one that’s the hardest to change.” - FY19_PT

### Sustainability

The “Sustainability” phase includes discussion of factors to support continued application of LEND concepts into daily service delivery, affecting children’s health and development across the lifespan (Edwards et al., 2014; Moullin et al., 2019). Trainees identified *mindset shifts* and *statewide connections* as drivers for sustained change, with suggestions for follow-up events and networking opportunities to enhance the effect of LEND training (Table 5). One provider describes the long-term effect of LEND training as:

**Table 5:** Perceived sustainability of the effect of LEND training among long-term trainees

Theme	Illustrative Quotes
Mindset shifts	“My competence and my ability to say I’ve been trained, I was very present in that training, I took copious notes, I’ve had all this experience, I created a program for parents…I’m prepared.” - FY21_SW
Statewide network	“I’m still connected to a lot of the people that I met through the LEND program as well. Like we’re friends on Facebook, I know if I need to reach out and if I have any questions in regard to services, I know that I can still call those people today.” - FY19_SCHOOL
	“I feel like the limitations that my LEND cohort had with being all virtual were kind of a bummer. I learn better in person typically, and I feel like things even more unofficial networking…it’s so much easier in person to have a side conversation and introduce yourself…versus like you can’t really go off to the side in a Zoom chat.” - FY22_SCHOOL
	“I wish there was some kind of follow up I guess, even if it’s like once a year or twice a year where we can even just touch base with our cohort of LEND members, just to touch in, see what everybody is doing. See if there’s anything new we can learn or some professional ongoing development, to keep that connection going.” - FY19_SCHOOL

“My competence and my ability to say I’ve been trained, I was very present in that training, I took copious notes, I’ve had all this experience…I’m prepared.” -FY21_SW.

Many trainees sought interdisciplinary settings following LEND and described intermittently contacting fellow trainees for help with difficult cases. Those who moved from their residence as a LEND trainee—within or beyond state borders—reported difficulty maintaining and/or re-establishing multidisciplinary relationships. For some, COVID-19 hindered their ability to form lasting relationships within their cohorts. However, these individuals report anticipation of evolving relationships as a byproduct of their skills acquired during LEND training.

Most trainees suggested opportunities to better sustain LEND connections. These suggestions were reported across school and healthcare environments, including invitations to attend LEND events with current cohorts, intermittent in-person events for live networking with past and current trainees, and annual events to connect across cohorts.

## Discussion

Interdisciplinary healthcare provider education is a well-documented means to promote collaborative, team-based care across pediatric settings—a “best practice” standard for optimal patient outcomes (Beebe et al., 2018; Elgen et al., 2021; Fair et al., 2016; Rosenberg et al., 2015). As a well-established training program nationwide, LEND provides an opportunity to support providers’ in developing evidence-based approaches to practice, research, leadership, and advocacy and to integrate national priorities into practice to address significant care gaps among CYSHCN (Edwards et al., 2014; Rosenberg et al., 2015; Weber et al., 2020, 2021). Further, LEND partners with individuals with disabilities and their families, who are key to driving multi-level solutions for existing gaps (Kuo et al., 2022; McLellan et al., 2022). Still, this study’s findings suggest that LEND graduates are limited in implementing and maintaining the effect of their training due to time and financial constraints of the U.S. healthcare system (*outer contexts*) (Moullin et al., 2019). Aligned with the life course perspective, discrepancies in LEND trainees’ abilities to implement their training within the healthcare system may elicit lifelong implications for CYSHCN and their families (Bengtson & Allen, 1993; Edwards et al., 2014).

Existing research agendas suggest that providers are often molded to fit systemic needs, rather than a healthcare system that is reflective of high quality care for CYSHCN and their families (Coller et al., 2020; Kuo et al., 2022; McLellan et al., 2022). While family-centered, multidisciplinary care within a medical home is well-documented as best practice, interviewees repeatedly reported that their graduate training was insufficient, compared to the translational skill development acquired through LEND (Beebe et al., 2018; Elgen et al., 2021; Fair et al., 2016; Rosenberg et al., 2015). Nationwide, LEND long-term trainees are often clinical fellows and early clinicians, who are eager to develop their leadership skills and set their career trajectories. LEND trainees discussed significant improvements in their service delivery and patient outcomes, largely attributed to acquiring an adjusted posture of humility and empathy for patients and their families as well as improvements in communication, confidence, and leadership skills within their local networks. However, trainees repeatedly identified systemic barriers, including waitlists, insurance limitations, and allotted time-per-patient, that prevent trainees’ from fully integrating their LEND training into practice. The systemic barriers reported by providers are not unique to this state and identify an opportunity for trainees from 60 LEND programs nationwide to band together, implement evidence-based strategies, and advocate for systems-level change across the U.S. healthcare system.

Trainees provided detailed examples of patient-level advocacy, from addressing parent concerns of a CYSHCN who identified as non-Hispanic Black and potential interactions with law enforcement to connecting families with community-based resources for well-rounded support. Still, trainees infrequently mentioned advocacy training or examples beyond the patient-provider relationship or local networks. One trainee in the sample described her involvement with a national-level advocacy board for transparent payment models, with hopes of making healthcare costs visible to patients before receiving treatment and adapting insurance-related restrictions for providers. With cohorts of passionate healthcare providers nationwide, LEND may consider building on leadership training and advocating for large-scale change through a massive provider network at the front lines of clinical care. The healthcare system has demonstrated pioneering resiliency in recent years to meet patient needs and LEND has a sensitive opportunity to emphasize advocacy training—driven by providers, policymakers, individuals with disabilities, and their families (Coleman et al., 2022; Geweniger et al., 2022; McLellan et al., 2022). The integration of a national framework with priority care gaps intended to serve a population these trainees are passionate about provides a key opportunity to shape the future research, practice, and policy of pediatric medicine in the US.

### Key Takeaways Related to EPIS Framework

LEND trains healthcare professionals to become leaders within their disciplines and settings. With foundational training, healthcare providers experience a dynamic relationship between their practice setting and implementation of their training components with continual adaptations for inner and outer contexts. LEND offers an opportunity for healthcare providers to develop leadership skills (*inner context*) and is woven into organizational characteristics and staffing processes. Trainees’ provider-level interactions are influenced by leadership within their practice setting, systemic limitations (*service environment/policies*), funding of services (*insurance*), and advocacy opportunities for CYSHCN and their families to access high-quality healthcare services. With established community-academic partnerships and expert leadership among LEND faculty, LEND may consider emphasizing state- and national-level advocacy and further engaging legislators, policymakers, and lawmakers in their curriculum nationwide.

### Recommendations for LEND

To facilitate sustainability of the effect of LEND training, providers requested increased follow-up from LEND faculty and opportunities for in-person networking. Several LEND programs transitioned to virtual programming following the onset of COVID-19; however, LEND faculty continue to seek balance in the hybrid format to maintain accessibility for working professionals while offering in-person events. Additionally, providers reported reliance on LEND resources since program completion to sustain the effect of their training. However, in this state, LEND trainees lose access to the virtual platform following program completion and a few trainees requested sustained access to resources. National LEND programs may develop a semi-public, virtual platform for current and past LEND trainees to gather resources and promote evidence-based practices. As suggested by this study’s LEND faculty, long-term access to such resources may allow providers to continually engage in LEND beyond the duration of their enrollment year.

### Strengths and Limitations

This study represents a homogeneous population due to the recruitment methods and demographic characteristics of this population. In addition, this study’s lead author is a LEND graduate from 2018 to 2022. However, member checking and multiple coders were employed to reduce these biases. This study intends to serve a baseline for implementation of *Blueprint for Change* among interdisciplinary healthcare providers nationwide.

The strengths of this study include its even distribution of participants by training year and by discipline, reducing bias in the results of this multi-year evaluation. Further, LEND leadership—including the director and a founding faculty member—participated in member checking to challenge and validate this study’s findings. These findings were shared with the LEND leadership team to inform program objectives.

## Conclusions

While LEND has the potential to advance large-scale agendas for pediatric healthcare reform, trainees are not immune to systemic limitations of U.S. healthcare systems. To optimize the effect of LEND training, multi-level, systemic adaptations must allow providers to follow best practice guidelines, as evidenced in existing research and policy (Coller et al., 2020; Hoover et al., 2022; Kuo et al., 2022; McLellan et al., 2022). With service delivery improvements, as described in this study’s findings, CYSHCN and their families may experience improvements in health outcomes, well-being, and quality of life.

## Electronic Supplementary Material

Below is the link to the electronic supplementary material.

Supplementary Material 1

## Data Availability

Not applicable.

## Abbreviations

LEND: Leadership in Neurodevelopmental and Related Disabilities
MCHB: Maternal and Child Health Bureau
EPIS: Evaluation, Preparation, Implementation, and Sustainment framework
CYSHCN: Children and youth with special health care needs

## Code Availability

This study’s codebook is included in the Appendices.
