# Supplementary material for: Supporting the Advancement of a National Agenda for Pediatric Healthcare Reform: A multi-year Evaluation of a Leadership Education in Neurodevelopmental and Related Disabilities Program
Source: Matern Child Health J. 2025 Jan 23;29(2):205–16. doi: 10.1007/s10995-025-04040-7 (PMC11821710; doi:10.1007/s10995-025-04040-7)
Supplement: Supplementary file 1 — Supplementary Material 1 [file 10995_2025_4040_MOESM1_ESM.docx]

**Appendices**

**Appendix A: Interview Guide**

*Introduction*

- Tell me about your current role. (Probe(s): provider type, setting, etc.)
  - Is this the same position you held when you were a LEND trainee?
    - If not, did LEND influence this position change? Tell me more about that.
- If you were talking to a provider who had not heard of LEND before, how would you describe this program?

*Measurements*

- How do you use your LEND training in your daily practice? Tell me more about that.
  - Probes: equity, advocacy, partnership
- What changes have you made in your service delivery as a result of being a LEND trainee?*,**
  - Probe: Has this had an impact on your organization/site’s service delivery?
  - How have you been supported (or not supported) in making these changes in your practice setting?
  - How difficult (or not difficult) was it for you to implement these changes in your daily practice?
    - How do you feel the quality of your services has changed as a result of your involvement in LEND?**
- As a provider/clinician, how do you believe you could improve the accessibility of your services for CYSHCN and their families?**
  - What current system does your practice setting have in place to help CYSHCN and their families access necessary healthcare services?**
    - How do you think this process could be improved?*,**
      - How has LEND influenced on your perspective/awareness of this?
- How have your patients’ (CYSHCN) outcomes changed since you were a LEND trainee?*
  - Probes: quality of life, health outcomes, SDOH
  - How do you think your LEND experience influenced these changes?
    - How do you think having more provider experience influences improved outcomes among CYSHCN, compared to the influence of LEND?*
  - How have parents/caregivers/family members reacted to your service delivery changes?*

*Sustainability*

- What challenges have you faced in maintaining/sustaining the changes you’ve made in response to your LEND training in your daily practice?
- What has made it easier for you to maintain the changes you’ve made in response to your LEND training “tools” in your daily practice?
  - Probes: “tools” = tactics/approaches
- What changes could be made to the LEND curriculum that would have better supported you long-term as a clinician/provider?
  - Probe: How would this make an improvement? Tell me more about what you mean by that.

Notes:

*Denotes questions related to “Critical Area 2: Family and child well-being and quality of life” (McLellan et al., 2022)

**Denotes questions related to “Critical Area 3: Access to Services” (McLellan et al., 2022)

**Appendix B: Initial Codebook for Data Analysis**

EPIS Codebook, based on operationalization in existing literature (Moullin et al., 2019)

| Category | Labels | Code definition |
| --- | --- | --- |
| Outer context | Service environment/policies | State and federal sociopolitical and economic contexts that influence the process of implementation and delivery/use of the innovation  Ex. policies, legislation, mandates, auditing, monitoring and review |
|  | Funding/contracting | Fiscal support provided by the system in which implementation occurs. Fiscal support can target multiple levels (e.g., staff training, fidelity monitoring, provision of the innovation/EBP) involved in implementation and delivery/use of the innovation  Ex. Contracting arrangements; grants; fee-for service, addition to formulary; capitation fees, incentives |
|  | Interorganizational environment and networks | Relationships of professional organizations through which knowledge of the innovation/EBP is shared and/or goals related to the innovation/EBP implementation are developed/established  Ex. Inter-organizational collaboration, commitment, competition, co-opetition |
|  | Patient/client characteristics* | Demographics and individual characteristics of the target population/end user  Exs. Socioeconomic status, health condition, comorbidities, age, gender, motivation |
|  | Patient/client advocacy* | Support or marketing for system change based on consumer needs, priorities and/or demographics  Ex. Client advocacy; class-action lawsuits, consumer organizations |
| Innovation factors* | Innovation/EBP developers | Characteristics of the individuals or team(s) responsible for the creation of the EBP/innovation that may be the subject of implementation efforts  Exs. Engagement in implementation, continuous quality improvement, rapid-cycle testing, prototyping |
|  | Innovation/EBP Characteristics | Features or qualities of innovations to be implemented  Exs. Complexity, ease of learning, cost, burden, reporting requirements |
|  | Innovation/EBP fit* | The extent to which the innovation/EBP fits the needs of the population served or context in which it is implemented  Exs. Innovation/EBP structural and process fit with system, organizations, providers, patients/clients |
| Bridging factors* | Community/academic partnerships* | Active partnerships between researchers and key community stakeholders, who can represent multiple levels involved in implementation (e.g., system representatives, organizational leaders, providers, consumers), that can facilitate successful implementation and delivery/use of the innovation  Exs. Community participation; partnerships; ongoing positive relationships; valuing multiple perspectives |
|  | Purveyors/intermediaries | Organizations or individuals providing support or consultation for implementation and/or training in the innovation  Exs. Implementation readiness assessment, strategy development, training support |
| Inner Context | Organizational characteristics | Structures or processes that take place and/or exist in organizations that may influence the process of implementation  Exs. Culture; climate; readiness for change; structure; leadership; receptive context; absorptive capacity; social network support |
|  | Leadership | Characteristics and behaviors of individuals involved in oversight and/or decision-making related to EBP implementation within an organization  Exs. Competing priorities; use of climate/culture embedding mechanisms; transformational leadership; implementation leadership |
|  | Quality and fidelity monitoring/support* | Processes or procedures undertaken to ensure adherence to active delivery of the innovation/EBP and/or an implementation strategy  Exs. Fidelity support system; quality assurance evaluation; continuous quality improvement |
|  | Organizational staffing processes | The processes or procedures in place at an organization related to the hiring, review, and retention of staff involved in the active delivery of the innovation/EBP and/or its implementation  Exs. Professional training and qualification related to EBI delivery; staff turnover |
|  | Individual characteristics | Shared or unique characteristics of individuals (e.g., provider, supervisor, director) that influence the process of implementation  Exs. Attitudes towards EBP; demographics and/or background; client characteristics; job demands |
| Stages of EPIS | Exploration | In the Exploration phase, a service system, organization, research group, or other stakeholder(s) consider the emergent or existing health needs of the patients, clients, or communities and work to identify the best EBP(s) to address those needs, and subsequently decides whether to adopt the identified EBP. In addition, consideration is given to what might need to be adapted at the system, organization, and/or individual level(s) and to the EBP itself |
|  | Preparation | In the Preparation phase, the primary  objectives are to identify potential barriers and facilitators of implementation, further assess needs for adaptation, and to develop a detailed implementation plan to capitalize on implementation facilitators and address potential barriers. Critical within the Preparation phase is planning of implementation supports (e.g., training, coaching, audit, and feedback) to facilitate use of the EBP in the next two phases (Implementation and Sustainment) and to develop an implementation climate that indicates that EBP use is expected, supported, and rewarded |
|  | Implementation | In the Implementation phase and guided  by the planned implementation supports from the Preparation phase, EBP use is initiated and instantiated in the system and/or organization(s). It is essential that ongoing monitoring of the implementation process is incorporated to assess how implementation is proceeding and adjust implementation strategies to support efforts accordingly |
|  | Sustainment | In the Sustainment phase, the outer and  inner context structures, processes, and supports are ongoing so that the EBP continues to be delivered, with adaptation as necessary, to realize the resulting public health impact of the implemented EBP. |

*Represents factors that are new or adaptations based on the original EPIS 2011 paper

Codebook of *Blueprint for Change* Operationalization(Brown et al., 2022; McLellan et al., 2022)

| Category | Labels | Code definition |
| --- | --- | --- |
| CA 1: Health equity  *Vision: All CYSHCN have a fair and just opportunity to be as healthy as possible and thrive throughout their lives (e.g., from school to the workforce) without discrimination and regardless of the circumstances in which they were born or live.* | Principle 1 | Structural and systemic causal barriers to health equity are eliminated, including discrimination, poverty, and other social risk factors. |
|  | Principle 2 | Sectors, systems, and programs that fund, deliver, and monitor services and supports for CYSHCN are designed and implemented to reduce health disparities and improve health outcomes for all CYSHCN. |
| CA 2: Family and child wellbeing and quality of life  *Vision: The service system prioritizes quality of life, well-being and supports flourishing for CYSHCN and their families.* | Principle 1 | Families, regardless of circumstance, can access high-quality, affordable, community-based services that support the medical, behavioral, social, and emotional well-being of the child or youth and whole family. |
|  | Principle 2 | Health systems place value on the measurement and use of both child and family well-being and quality-of-life outcomes, and health outcomes. |
| CA 3: Access to services  **Vision:***CYSHCN and their families have timely access to the integrated, easy-to-navigate, high-quality health care and supports they need, including but not limited to physical, oral, and behavioral health providers; home and community-based supports; and care coordination throughout the life course. This critical area recognizes the educational system as an entry point and major deliverer of services for children and families.* | Principle 1 | All services and supports at the individual, family, community, and provider levels are easy for families and professionals to navigate when, where, and how they need them. |
|  | Principle 2 | The workforce is trained to meet the needs of CYSHCN and their families, reflects the families and communities they serve, and is culturally responsive. |
|  | Principle 3 | Service sectors increase the ability of CYSHCN and their families to access services by addressing administrative and other processes that hinder access |
| CA 4: Financing of services  **Vision:***Health care and other related services are accessible, affordable, comprehensive, and continuous; they prioritize the well-being of CYSHCN and families.* | Principle 1 | Health care and other related services for CYSHCN and families are financed and paid for in ways that support and maximize an individual’s values and choice in meeting needs. |
|  | Principle 2 | Health and social service sector investments address social determinants of health to increase family well-being and flourishing. |
|  | Principle 3 | Payers and service sectors adopt value-based payment strategies that support families, advance equity, and incorporate continuous quality improvement by enhancing team-based integrated care. |
